# Superparamagnetic Iron Oxide Nanoparticles Function as a Long-Term, Multi-Modal Imaging Label for Non-Invasive Tracking of Implanted Progenitor Cells

**DOI:** 10.1371/journal.pone.0108695

**Published:** 2014-09-24

**Authors:** Christina A. Pacak, Peter E. Hammer, Allison A. MacKay, Rory P. Dowd, Kai-Roy Wang, Akihiro Masuzawa, Bjoern Sill, James D. McCully, Douglas B. Cowan

**Affiliations:** 1 Boston Children's Hospital and Harvard Medical School, Department of Anesthesia, Boston, Massachusetts, United States of America; 2 University of Florida, Department of Pediatrics, Gainesville, Florida, United States of America; 3 Boston Children's Hospital and Harvard Medical School, Department of Cardiac Surgery, Boston, Massachusetts, United States of America; 4 Beth Israel Deaconess Medical Center and Harvard Medical School, Department of Surgery, Boston, Massachusetts, United States of America; Centro Cardiologico Monzino, Italy

## Abstract

The purpose of this study was to determine the ability of superparamagnetic iron oxide (SPIO) nanoparticles to function as a long-term tracking label for multi-modal imaging of implanted engineered tissues containing muscle-derived progenitor cells using magnetic resonance imaging (MRI) and X-ray micro-computed tomography (μCT). SPIO-labeled primary myoblasts were embedded in fibrin sealant and imaged to obtain intensity data by MRI or radio-opacity information by μCT. Each imaging modality displayed a detection gradient that matched increasing SPIO concentrations. Labeled cells were then incorporated in fibrin sealant, injected into the atrioventricular groove of rat hearts, and imaged *in vivo* and *ex vivo* for up to 1 year. Transplanted cells were identified in intact animals and isolated hearts using both imaging modalities. MRI was better able to detect minuscule amounts of SPIO nanoparticles, while μCT more precisely identified the location of heavily-labeled cells. Histological analyses confirmed that iron oxide particles were confined to viable, skeletal muscle-derived cells in the implant at the expected location based on MRI and μCT. These analyses showed no evidence of phagocytosis of labeled cells by macrophages or release of nanoparticles from transplanted cells. In conclusion, we established that SPIO nanoparticles function as a sensitive and specific long-term label for MRI and μCT, respectively. Our findings will enable investigators interested in regenerative therapies to non-invasively and serially acquire complementary, high-resolution images of transplanted cells for one year using a single label.

## Introduction

Non-invasive imaging techniques can provide important information about the retention and distribution of transplanted cells in experimental therapeutic trials. To acquire useful imaging data, it is essential to label these cells with a substance that provides the necessary contrast to enable their identification in whole animals or specific organs long after their transplantation. While many contrast agents have been developed for use with individual imaging systems, numerous situations exist in which it would be beneficial to employ one agent to provide contrast detectable by multiple modalities [1]. Several research groups have created or modified compounds for this purpose [2], [3], [4], [5], [6], [7]; however, the utilization of colloidal superparamagnetic iron oxide (SPIO) particles coated with dextran as a reliable, well-characterized, and readily-available contrast agent for long-term tracking of transplanted cells using both magnetic resonance imaging (MRI) and micro-computed tomography (μCT) has not been described. In view of that, we evaluated the use of SPIO nanoparticles as a multi-modal contrast agent to identify progenitor cells within engineered tissues implanted in the atrioventricular (AV) groove of Lewis rat hearts for six months and one year.

For over a quarter of a century, the extremely high relaxivity of iron oxide particles has been exploited to provide strong contrast in MRI applications [8], [9], [10], [11]. While SPIOs were originally used for diagnostic purposes, a number of studies demonstrated the ability of these particles to non-invasively identify and track transplanted cells by MRI [12], [13], [14], [15]. One key attribute of SPIO particles is that they are biologically inert and believed to safely degrade via normal iron recycling pathways when released from dying cells [16]. Another beneficial feature is that cells can be heavily-labeled with SPIOs and remain viable without affecting their proliferative capacity [17]. On the other hand, the intracellular concentration of SPIO particles can be diluted by cell division resulting in eventual loss of MRI signal [18]. Other problems associated with their utilization in cell tracking studies include the ingestion and removal of labeled cells from target tissues by macrophages and the persistence of large deposits of iron particles released from dead and dying transplanted cells [19].

Although it is well established that MRI enables non-invasive tracking of SPIO-labeled cells with high spatial resolution and sensitivity as well as superb soft tissue detail, there are few, if any, studies that evaluate the efficacy of this label beyond a few days or weeks. In published reports using iron-labeled cells transplanted to the myocardium, hypointense signals detected by MRI commonly dissipate over time, which is largely attributable to poor survival and retention of the transplanted cells [20]. The inability to track SPIO-labeled cells in the heart over long periods of time is likely predominantly related to transplanted cell loss as a result of the cell delivery method and the host tissue micro-environment rather than the depletion of signal because of immune reactions, iron metabolism, or dilution from cell proliferation [21], [22]. In contrast, previous studies from our laboratory have demonstrated the viability and survival of muscle-derived progenitor cells incorporated within engineered tissue constructs for well over two years following implantation in the rat heart [23]. As a result, we presumed our rodent model may be better suited to evaluate the long-term imaging potential of SPIO-labeled cells using magnetic resonance-based methods.

SPIO particles are superb contrast agents for MRI because of their ability to shorten transverse relaxation times (T_2_ and T_2_*), leading to increased signal intensity in target tissues. They also permit serial tracking of transplanted cells; however, there are several limitations to imaging these types of compounds. For instance, labeled regions can be difficult to distinguish from other areas of negative contrast caused by water-fat interfaces and blood flow. Moreover, a quantitative assessment of implanted cells cannot be performed on very hypointense images and the signal intensity tends to over-estimate the number of transplanted cells due to the exceptionally high sensitivity of MRI. Consequently, we wanted to identify other non-invasive imaging modalities that would complement the sensitivity of MRI-based cell detection with more precise localization of SPIO-labeled cells. Given that several imaging techniques have been developed to suppress the well-documented X-ray computed tomography imaging artifacts caused by iron-containing metal implants [24], [25], we reasoned that SPIO nanoparticles could be identified using μCT. SPIO nanoparticles imaged by this technique would generate positive contrast images, which would be less prone to misinterpretation when compared to the large amount of negative contrast often detected by MRI. Because of the anticipated lower sensitivity of this imaging method, μCT may also avoid the problem of over-estimating the number of transplanted cells as is the case with T_2_- and T_2_*-weighted MRI. Accordingly, we investigated whether the combination of MRI and μCT can provide superior information about the number and position of transplanted iron-labeled cells than either imaging technique alone.

In this report, we show SPIO nanoparticles function as a long-term label of transplanted cells in the heart using two complementary imaging modalities. We found MRI to be a more sensitive technique and μCT was more precise for detection of SPIO-labeled cells; however, the combination of these non-invasive imaging methods provides a powerful new approach to track transplanted cells in preclinical animal models over extended periods of time without the need for genetic manipulation of the delivered cells.

## Methods

### Myoblast Isolation and Labeling with SPIO Nanoparticles

Primary skeletal myoblasts were isolated from day 2 (d2) neonatal rats essentially as described [26]. Feridex IV (ferumoxides injectable solution) (Berlex) was used to label cells as previously described [13]. Briefly, for every 1.5×10^7^ cells to be labeled, 27 µg Poly-L-lysine (PLL) hydrobromide (Sigma) and 0 µg, 0.37 µg, 1.48 µg, 2.30 µg, 4.66 µg, 9.33 µg, 18.67 µg or 37.3 µg of SPIO particles (*i.e.* Feridex IV) was added to 9 mL of serum free Ham's F-10 culture medium (Thermo Scientific). The solution was mixed and incubated on a rocking platform at 25°C for 1 hour. The medium was removed from the culture plates and the cells were rinsed twice with phosphate buffered saline (PBS), pH 7.4. The PLL-SPIO mixture was then added dropwise to the cells and the plates were incubated at 37°C for 1 hour. The PLL-SPIO solution was aspirated and the cells were rinsed twice with PBS. Ham's F-10 medium containing 20% fetal bovine serum (FBS) (Atlanta Biologicals), 1% penicillin-streptomycin (Life Technologies), and 1% Fungizone (Life Technologies) was added to the cells and placed in a humidified 37°C incubator supplemented with 5% CO_2_. Accustain Iron Staining (Sigma) was performed on 4% paraformaldehyde (PFA)-PBS fixed cells attached to LabTEK slides representing each SPIO concentration. Three experiments were performed for each concentration and images were acquired using brightfield microscopy.

### Incorporation of Cells within Fibrin Sealant

One day after labeling with PLL-SPIO, cells were rinsed twice with PBS and then detached from the plates using 0.05% trypsin-EDTA (Life Technologies). For creation of SPIO gradient standards, approximately 3×10^7^ cells were collected by centrifugation and resuspended in 30 µL of PBS and mixed with 70 µL of the thrombin portion of TISSEEL fibrin sealant (Baxter). A 1 mL syringe was loaded with thrombin containing the cells. Another 1 mL syringe was loaded with 100 µL of the fibrinogen portion of TISSEEL fibrin sealant. The Duploject system (provided with TISSEEL) was used to simultaneously mix and eject the two solutions into a micro-centrifuge tube. Three tubes were generated and imaged for each SPIO concentration and three slices from each acquisition were averaged to collect intensity or opacity data. For injection into the AV groove of each rat heart, 3×10^7^ cells were resuspended in 30 µL of PBS and mixed with TISSEEL as described above. The Duploject system was used to eject the fibrin sealant into the AV groove of each rat's heart.

### Rat Model, Cell Implantation, and Heart Extraction

Experimental rat hearts (n = 6) were implanted with fibrin sealant containing SPIO-labeled cells, while control rats (n = 3) were implanted with TISSEEL containing unlabeled cells. Adult female Lewis rats were pre-anesthetized with 75 mg/kg Ketamine and 5 mg/kg Xylazine via intraperitoneal (IP) injection followed by intra-tracheal intubation with an appropriate sized intravenous (IV) catheter. Animals were connected to a small animal respirator (Harvard Apparatus INSPIRA) to deliver a mixture of 0.5% isoflurane and 99.5% oxygen. Respiration rate and stroke volume were adjusted based on bodyweight. An antero-lateral incision at the right thoracic wall was performed and the muscular planes were dissected. The thoracic cavity was entered through the 4^th^ intercostal space, the pericardium was opened, and the heart exposed. The AV groove was identified and the epicardium was removed on both the right atrium and the right ventricle to reveal the myocardium. A portion of the cell and fibrin sealant mixture was applied to this region. The pericardium and the chest wall were closed in layers using a monofilament absorbable suture (5-0 Prolene) and the incision was infiltrated with Bupivicaine. Pneumothorax was evacuated by insertion of an appropriate sized catheter. The animals were then extubated after weaning from the respirator and recovered under analgesia (0.5 mg/kg Buprenorphine sub-cutaneously every 12 hours for 3 days). Six months and one year later, the rats were subjected to MRI and μCT. Hearts were then excised, rinsed in PBS, perfusion-fixed in 4% paraformaldehyde and fixed at 4°C using 4% PFA in PBS. Isolated hearts were also imaged by MRI and μCT. Studies were carried out in accordance with the Guide for the Care and Use of Laboratory Animals of the National Institutes of Health and our animal protocol was approved by the Boston Children's Hospital IACUC.

### Magnetic Resonance Imaging

To acquire images and T2* relaxation times, the cell samples, Lewis rats, and excised hearts were placed in a BioSpec 70/30 USR 7T MRI System (Bruker) running ParaVision Version 5.1 software or a BioSpec 4.7T MRI system (Bruker) running ParaVision Version 4.0. Rats were anesthetized with 1.5–2.0% isoflurane at an oxygen flow of 1 L/minute via nose cone. Respiration was maintained between 38 and 55 breaths per minute and ECGs acquired throughout the acquisitions showed heart rates between 255 and 315 beats per minute. After an initial positioning scan, multiple-slice, FLASH cine images were acquired with the following parameters: For animals – pulse repetition time, 52.747 ms; echo time, 1.460 ms; field of view, 60×60 mm; slice thickness, 0.5–1.0 mm; acquisition matrix size, 256×256: For cell gradients and hearts – pulse repetition time, 300 ms; echo time, 2.4 ms; field of view, 60×60 mm; slice thickness, 0.5–1.0 mm; acquisition matrix size, 256×256. Images were reconstructed and intensity data was analyzed using ImageJ software (http://imagej.nih.gov/ij/download.html).

### Micro-Computed Tomography

Micro-CT was performed using an Albira Preclinical Imaging System (Bruker) running Albira Software Suite version 1.530. Excised hearts were scanned at an X-ray tube voltage and current of 45 kV and 400 µA, respectively, using 600 projections per scan. The reconstructed images were 512×512×512 voxels with an isotropic voxel size of 125 µm. SPIO gradient images were analyzed using the Amide software package (http://amide.sourceforge.net). Cross sectional and volume rendered images identifying iron-labeled cells in live rats and excised hearts were created using VolView, version 3.4 (Kitware).

### Histological Staining of Heart Tissue Sections

Excised hearts were retrograde perfused in the constant flow Langendorff mode at 22°C for 10 min with 4% PFA in PBS prior to passive fixation at 4°C overnight in the same solution. After paraffin embedding and sectioning (5 µm thickness), slides were baked for 1 hour at 65°C, deparaffinized in xylenes, rehydrated through a graded ethanol series, and subjected to antigen retrieval by heating three times for 5 min in 1 mM EDTA (pH 8.0) using a 700 W microwave oven set to high. The PBS solution was changed between each heating cycle. Slides were then used for either routine histological staining (Masson's trichrome or Prussian blue with pararosaniline) or immunohistochemical staining with the following antibodies: anti-sarcomeric α-actinin (ACTN) (Abcam ab68167), anti-macrophage (MAC387) (Abcam ab22506), or anti-cardiac troponin T antibody (cTnT) (Abcam ab8295). Some slides were fluorescently labeled with Alexa Fluor 488 phalloidin (Life Technologies). Primary antibodies were detected with species appropriate highly cross-adsorbed Alexa Fluor-conjugated secondary antibodies (Life Technologies), stained with 4′,6-diamidino-2-phenylindole (DAPI) (Life Technologies), and visualized with an Olympus FSX-100 fluorescent microscope.

### Statistical Analyses

The percentage of Prussian blue stained cells was determined by three blinded observers for each iron oxide concentration. For MRI and μCT detection of SPIO-labeled cells, three slices from each image acquisition were combined to collect mean T2* relaxation times and pixel intensity or voxel opacity data, respectively. Values were generated using SigmaPlot 12.5 statistical analysis software and significance was determined using a paired t-test. These values are reported as mean ± standard error of the mean and a P<0.05 was considered significant.

## Results

### Creation of a SPIO-Labeled Cell Gradient

**Figure 1:**
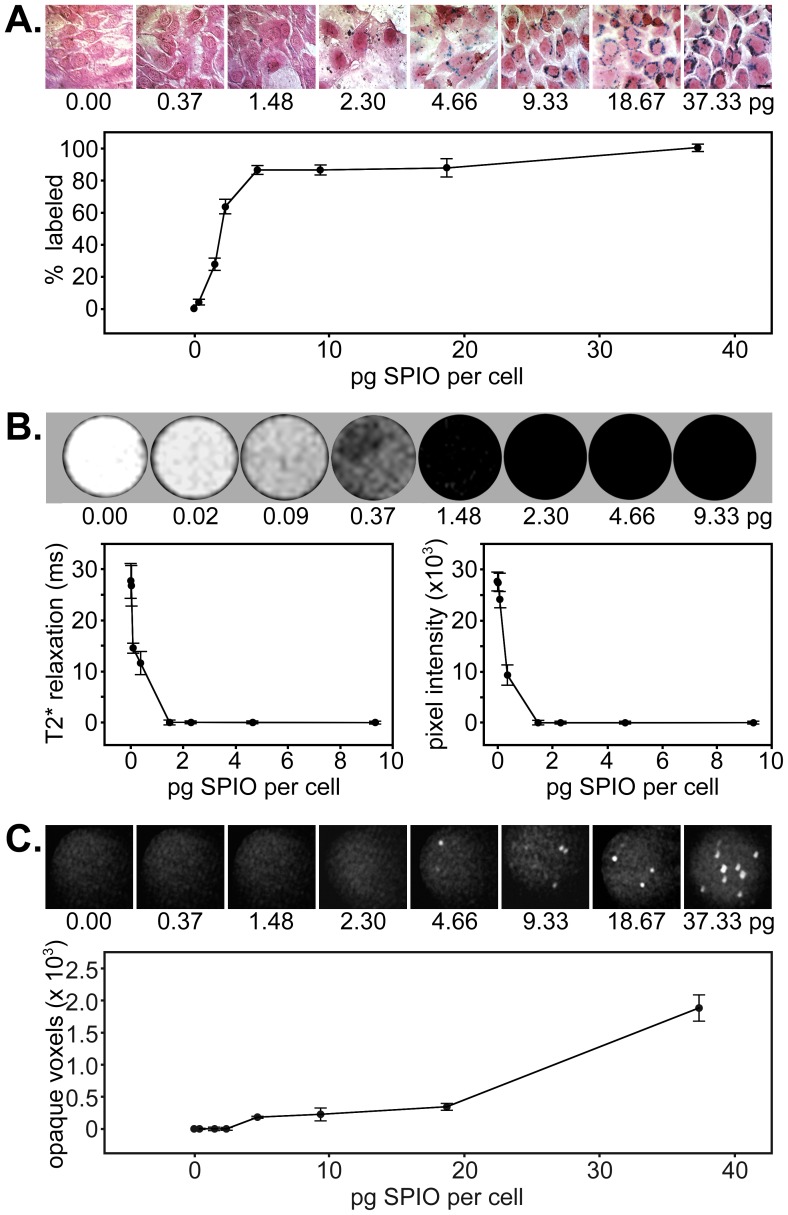
*In vitro* detection of SPIO nanoparticle-labeled muscle progenitor cells by histology, MRI, and μCT. A) Cells incubated with increasing concentrations of SPIO particles and PLL were fixed and stained with Prussian blue and pararosaniline to identify iron-labeled cells. Slides were imaged using brightfield microscopy. The graph shows the percentage of SPIO-labeled cells at each concentration (mean ± standard error of the mean) as determined by analysis from three blinded observers. B) MRI of SPIO-labeled cell standards incorporated within fibrin sealant in micro-centrifuge tubes. The graphs display T2* relaxation time versus SPIO concentration (left) and average pixel intensity versus SPIO concentration (right) as determined from mean values generated from three different labeling experiments. C) μCT images of the same SPIO-labeled cell standards incorporated within fibrin sealant as depicted in B). The graph displays the number of opaque voxels versus SPIO concentration as determined from values generated from three different labeling experiments. Data represents mean ± standard error of the mean for each.

Our initial experiments focused on determining the amount of Feridex reagent needed to efficiently label cells in culture and remain detectable by histology, MRI, and μCT. Accordingly, primary rat myoblasts were seeded onto microscope slides and labeled with increasing amounts of SPIOs. One day after labeling, the cells were fixed and stained for the presence of iron (Figure 1A). For each SPIO concentration, five images from three experiments were acquired and enumerated by blinded observers to establish the proportion of labeled cells. Data points for each concentration represent the mean percentage of labeled cells ± standard error of the mean. In this way, we determined approximately 80% of the myoblasts were labeled at the 4.66 pg per cell SPIO concentration and 100% of the cells were labeled at the 37.33 pg per cell SPIO concentration. SPIO concentrations>1.48 pg per cell showed P-values<0.01 when compared to unlabeled cells or those exposed to 0.37 pg per cell.

### Assessment of SPIO-Labeled Cell Standards by MRI

For each concentration of SPIO particles, a total of 3×10^7^ cells were labeled, mixed with fibrin sealant, and injected into micro-centrifuge tubes. The cells for each tube (using three tubes per concentration) were distributed evenly within the fibrin matrix to create a total volume of 200 µL. Cross sectional images of the cellular organization in these tubes and T2* relaxation times for those areas were acquired using MRI (Figure 1B). Escalating negative contrast (*i.e.* hypointensity) was observed to correspond with increases in the number of SPIO particles used to label cells in each tube. Our data showed complete saturation of signal by MRI in tubes containing cells labeled at a concentration of 1.48 pg SPIO particles or more per cell. As expected, T2* relaxation times and pixel intensities were closely correlated. For both T2* relaxation data as well as pixel intensity data, SPIO concentrations ≥1.48 pg per cell showed P-values<0.01 when compared to those cells exposed to concentrations ≤1.48 pg per cell. Data points for each concentration represent mean T2* relaxation (ms) ± standard error of the mean (left panel) or mean pixel intensity ± standard error of the mean (right panel).

### Assessment of SPIO-Labeled Cell Standards by μCT

The same SPIO standards used in the MRI study were imaged using μCT (Figure 1C). Using this imaging method, enhanced signal from iron oxide was manifest as regions of positive contrast (*i.e.* hyperintensity), which corresponded to the increasing SPIO particle concentrations in the cells in each tube. While opaque, positive contrast regions were detectable at the 4.66 pg and 9.33 pg per cell concentrations, the 18.67 pg concentration generated a signal that was more readily apparent and likely to be detectable *in vivo*. In contrast to MRI detection of SPIOs, μCT imaging identified only those cells that contain relatively large deposits of iron. Although this imaging modality was less sensitive to lower SPIO concentrations, it did appear to more precisely identify the location of heavily-labeled cells and resulted in a linearity of detection comparable to that observed for Prussian blue-stained cells (Figure 1A) when compared to MRI (Figure 1B). SPIO concentrations ≥4.66 pg per cell showed P-values<0.01 when compared to those cells exposed to concentrations ≤4.66 pg per cell. Data points represent mean number of opaque voxels ± standard error of the mean.

### MRI and μCT of Implanted SPIO-labeled Cells within Engineered Tissues

**Figure 2:**
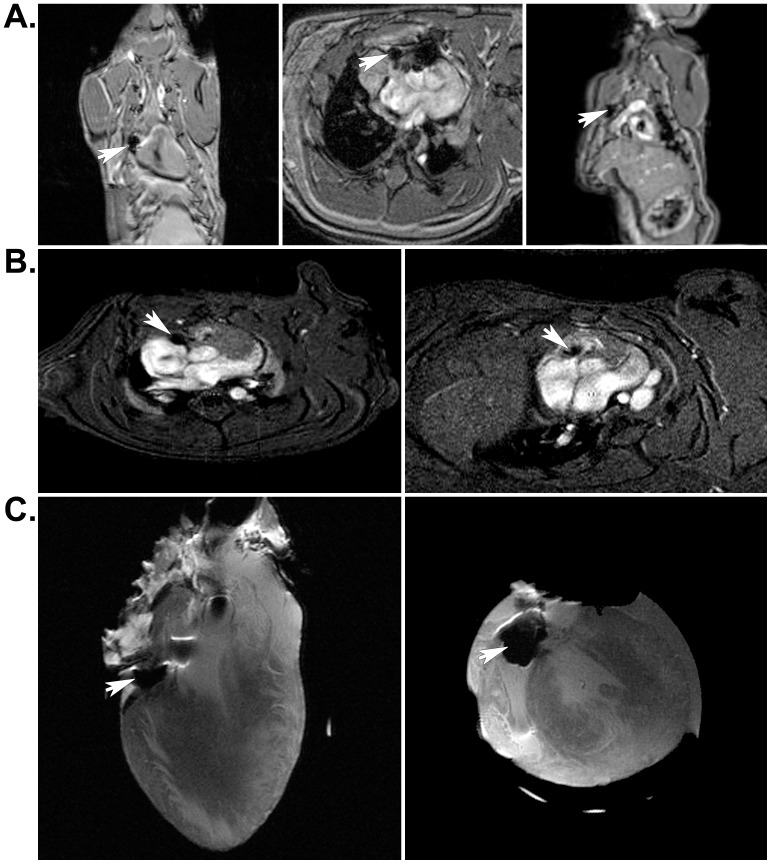
Localization of SPIO-labeled cells in implanted rat hearts by MRI. A) Arrows indicate the location of hypointense regions resulting from the presence of SPIO nanoparticles contained in the implant. Coronal (left), axial (center), and sagittal (left) MRI slices from *in vivo* cardiac imaging acquisitions were obtained 6 months post-implantation using a 7T magnet. B) Arrows indicate the location of SPIO-labeled cells (hypointense region) in short-axis slices from *in vivo* cardiac acquisitions taken from two different rats at 6 months and 1 year after surgical implantation of engineered tissues, respectively. These images were obtained using a 4.7T magnet. C) Arrows indicate the location of iron-labeled cells within the implant in an isolated heart that had been implanted with engineered tissues for 1 year. These coronal (left) and axial (right) MRI slices correspond to the *in vivo* images shown in A).

**Figure 3:**
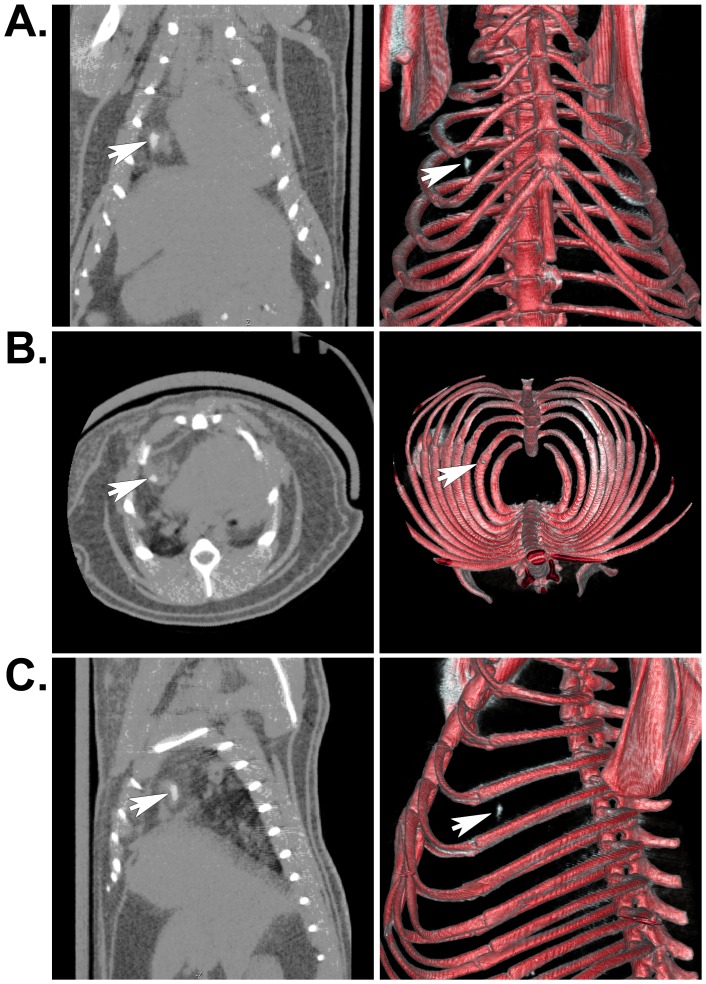
Localization of SPIO-labeled cells in whole animals by μCT. A) Arrows indicate the location of the SPIO nanoparticle-labeled muscle progenitors contained within engineered tissues that were implanted in the AV groove of an adult rat heart for 1 year. Unlike MRI images, the iron presents as a positive contrast or hyperintense signal. Coronal (A), axial (B), and sagittal (C) μCT images are shown. The panels on the left show individual μCT image slices, while the panels on the right depict the corresponding volumetric renderings from all of the acquired slices. These renderings were set to a threshold level that permitted simultaneous visualization of bone and iron-labeled cells. This processing resulted in loss of some soft tissue detail.

**Figure 4:**
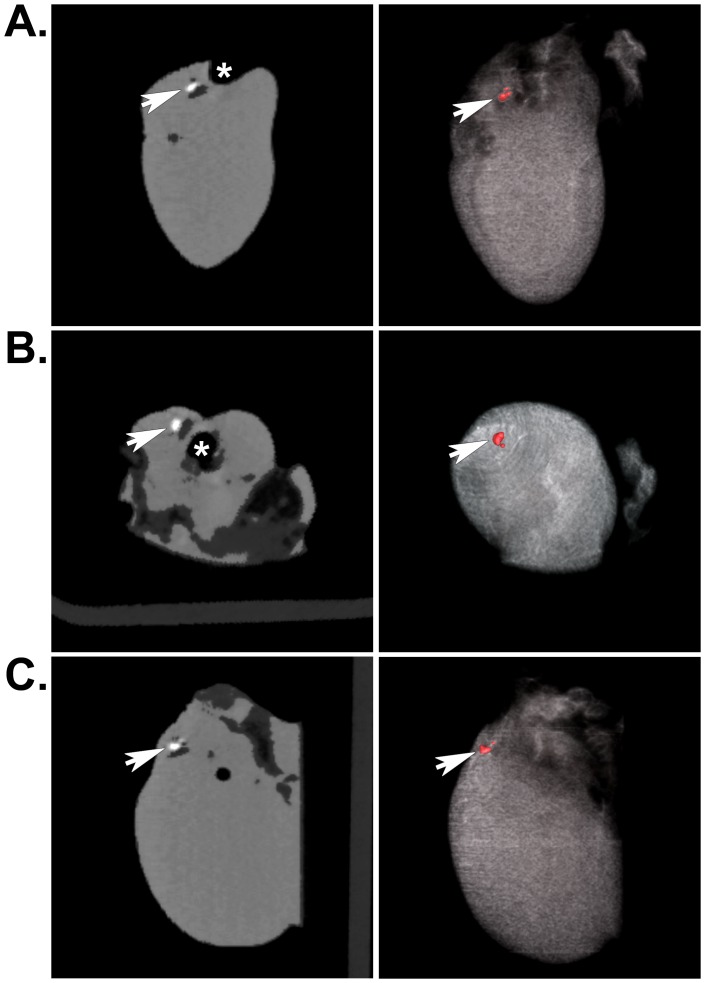
Localization of SPIO-labeled cells in excised hearts by μCT. A) Arrows indicate the location of the SPIO nanoparticle-labeled muscle progenitors contained within engineered tissues that were implanted in the AV groove of an adult rat heart for 1 year. Signal from iron is shown as a positive contrast signal (white spot). The asterisk indicates the position of the aorta exiting the heart. Coronal (A), axial (B), and sagittal (C) μCT images are shown. Once again, the panels on the left show individual image slices and the panels on the right show corresponding volumetric renderings. In the absence of signal from bone, soft tissue detail becomes more apparent.

Two days after isolation from neonatal rat skeletal muscle, myoblasts were incubated with 18.67 pg of SPIO particles per cell, mixed with fibrin sealant, and injected into the AV groove of rat hearts. Six months and one year after implantation, live animals or isolated hearts were assessed *in vivo* using each non-invasive imaging modality. As expected, MRI revealed hypointense regions where the SPIO-labeled cells were located in live rats (Figure 2A and B) (Movie S1 and S2) or in excised, fixed hearts (Figure 2C) using either a 4.7T or 7T magnet for acquisition. μCT imaging revealed positive contrast regions indicating the location of heavily-labeled cells, which was clearly noticeable in both cross sectional images and volumetric renderings from the *in vivo* acquisition from live rats 6 months after implantation surgery (Figure 3, A to C) (Movie S3) as well as from isolated hearts one year post-implantation (Figure 4A–C) (Movie S4). Control hearts did not show any equivalent hypointense signals by MRI or hyperintense signals by μCT (not shown). These signals were only noticeable in the experimental animals and excised hearts. MRI images were obtained using either a 4.7T or 7T magnet (Figure 2 B); however, there was no difference in the results acquired from either system.

### Histology of SPIO-labeled Cells in Implanted Engineered Tissues

**Figure 5:**
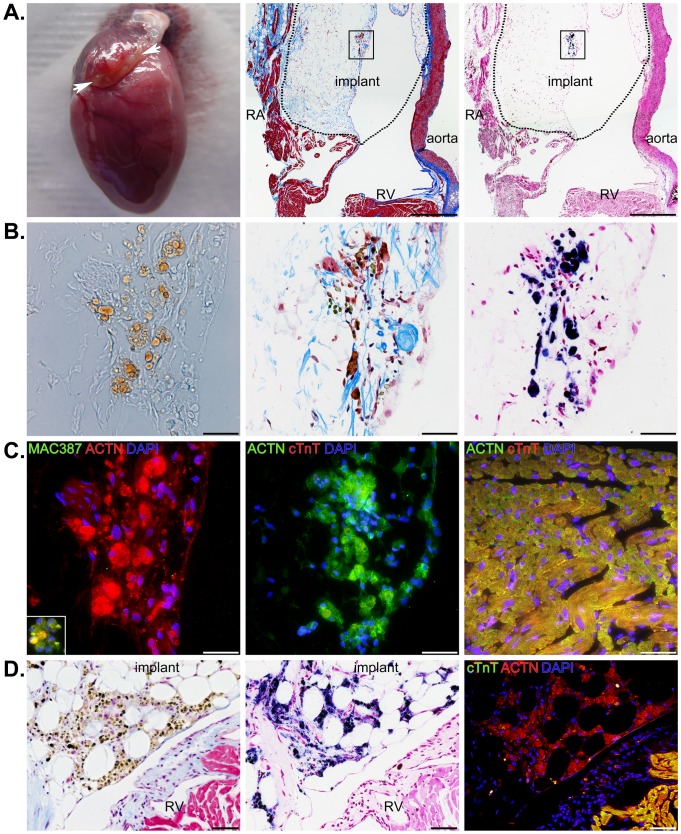
Localization of SPIO-labeled cells within the area of implants by histology. A) A photograph depicting the fibrin sealant-based implant in a heart excised one year after implantation (left panel), a low magnification image of a Masson's trichrome stained heart section (center panel), and a low magnification image from an adjacent serial section showing Prussian blue iron stain (right panel) of SPIO-labeled cells within the implant. The right atrium (RA), right ventricle (RV), and aorta are depicted along with an outline of the implant (dotted line). Scale bars equal 500 µm. B) Higher magnification brightfield images from the boxed region in A) showing an unprocessed serial section demonstrating iron (brown) can be detected in the cells of the implant and the same Masson's trichrome and Prussian blue stained sections from A). Scale bars equal 50 µm. C) The same section as the left panel from B) immunostained for the presence of macrophages (MAC387 in green), DNA (DAPI in blue), and striated muscle (ACTN in red) (left panel). Adjacent serial sections were immunostained for cardiac troponin T (cTnT in red), DNA (DAPI in blue), and striated muscle (ACTN in green) (middle panel). The right panel shows an image of ACTN, cTnT, and DAPI staining in the right ventricle. Scale bars equal 50 µm. D) Serial sections from a different heart stained for Masson's trichrome (left panel), Prussian blue with pararosaniline (middle panel), and immuno-fluorescence from secondary antibodies detecting binding of cTnT and ACTN antibodies in addition to DAPI staining of DNA. Iron is abundant in implanted cells, but is not apparent in the epicardium or myocardium of the right ventricle. The location of SPIO-labeled cells corresponds to the location of ACTN positive (red), cTnT negative (green) cells. Scale bars equal 50 µm.

The fixed hearts (Figure 5A, left panel) were embedded in paraffin, sectioned coronally, and stained with Masson's trichrome to distinguish cells (red) from connective tissue (blue) (Figure 5A, middle panel). Closer inspection of Masson's trichrome stained sections revealed that iron-labeled cells (greenish-brown) could be identified in the area of the implant. This observation was confirmed using Prussian blue and pararosaniline staining of adjacent serial sections (Figure 5A right panel). Higher magnifications of the implanted region identified in Figure 5A showed the vast majority of iron staining was confined to the myoblast cytoplasm (Figure 5B). A few rare deposits of iron appeared outside the myoblast plasma membrane and likely represent SPIO particles that were never internalized by the cells. We also found brightfield illumination alone was able to identify heavily-labeled cells (Figure 5B, left panel) and the presence of iron was confirmed in serial sections stained with Masson's trichrome and Prussian blue (Figure 5, middle and right panels). The heart sections in Figure 5 (A to C) correspond to the MRIs in Figure 2 (A and C) and the μCT images shown in Figures 3 and 4. Although we imaged (Figure 2B) and histologically stained all of the other hearts (Figure 5D), the combination of MRI, μCT, and histology from this particular heart demonstrated the sensitivity and precision of the two imaging modalities in identifying and localizing a small cluster of iron-labeled cells.

To confirm the labeled cells in tissue sections represented viable myoblasts and to look for the presence of infiltrating macrophages, we stained serial sections with MAC387, ACTN, and cTnT along with DAPI (Figure 5C). We found very few macrophages in the implants; though, some macrophages were identified in other regions of the heart (Figure 5C, left panel inset). There was no evidence at one year of macrophage ingestion of SPIO nanoparticle-labeled myoblasts. As expected, labeled cells did stain with the general muscle marker ACTN, but did not stain for cardiac-specific cTnT (Figure 5C, left and middle panels). For comparison, a region of right ventricular myocardium stained for both ACTN and cTnT (Figure 5C, right panel). In a different heart, we found large numbers of iron-labeled cells that were restricted to the region of the implant and showed a similar staining pattern to that described above (Figure 5D). Therefore, our histological analyses confirmed the presence of SPIO nanoparticle-labeled, skeletal muscle-derived cells in the expected area based on observations *in vivo* and in excised hearts using MRI and μCT one year following implantation.

**Figure 6:**
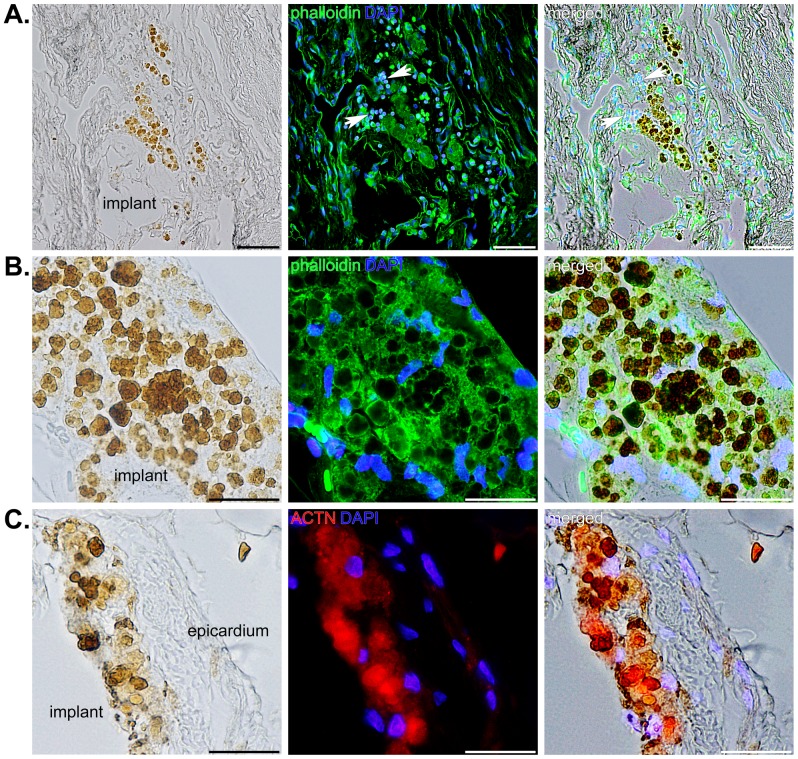
SPIO nanoparticle-labeled cells located within the implant in three different hearts. A) Brightfield, fluorescence, and merged images showing iron deposits in progenitor cells contained within the implant (left panel), staining of filamentous actin with phalloidin (green) and DNA (blue) (middle panel) in the same section, and the merged image (right panel). Arrows indicate a few inflammatory cells that are not positive for iron. Scale bars equal 50 µm. B) A different heart showing heavily-labeled cells in the implant using the same staining as described for A). Scale bars equal 25 µm. C) Iron was also evident (brown) within a portion of the implant immediately adjacent to the right ventricular epicardium in a separate tissue section (left panel) that was immunostained for ACTN (red) and DAPI (blue) (middle panel). The right panel shows an overlay of the two images to the left. Scale bars equal 25 µm.

Evaluation of over 400 slides from each of the implanted hearts showed similar results, while control hearts showed no indication of the presence of iron. The SPIO particles were obvious within many of the skeletal muscle-derived cells one year after implantation (Figure 6). A few cells that appeared to be macrophages were identified in one section; however, these infiltrating cells did not overlap with SPIO-labeled myoblasts (Figure 6A, arrows). Higher magnification images of heavily-labeled cells showed that iron deposits identified by brightfield illumination overlapped with filamentous-actin staining by phalloidin and, in some instances, large iron deposits appeared to displace the cytoskeleton (Figure 6B). Nevertheless, the larger iron deposits that accounted for the signals acquired by MRI and μCT always appeared to be contained in viable cells that stained for ACTN and were never found outside the region of the implant (Figure 6C).

## Discussion

While the use of SPIO nanoparticles as a highly effective contrast agent for MRI has been reported by numerous groups, their use as a cellular label for computed tomography has only been demonstrated by a few other laboratories at early time points (hours to days) [5] [27]. Since methods have been developed to suppress computed tomography acquisition artifacts in humans with iron-containing implantable medical devices [24], [25], it is not especially surprising to find that SPIO nanoparticles can be identified by μCT. For this reason, we suspected that the combination of MRI and μCT would offer complementary information for tracking cells *in vivo*. In the present study, we established that μCT enabled precise localization of iron-labeled cells in the heart, although this imaging modality was less sensitive than MRI. We also extended the effective imaging window to 1 year, which should allow for thorough assessment of preclinical, cell-based therapies directed at regenerating or repairing the heart.

In addition to identifying the amount of SPIO nanoparticles necessary for transplanted cell visualization by MRI and μCT, we also determined the detection limits of iron within labeled cells by histology (Figure 1). Our experiments established the number of SPIO nanoparticles necessary to completely label muscle-derived cells in culture. Similar to the findings of other investigators, we observed no apparent loss of cellular proliferative capacity or viability, even in heavily-labeled myoblasts [18], [28]. Using MRI, a SPIO concentration of 1.48 pg per cell so dramatically increased the proton relaxivity of the entire 200 mm^3^ volume of cellularized fibrin sealant that the pixel intensity was at a maximum (*i.e.* black). By comparison, μCT provided much more precise information regarding the location of heavily-labeled cells and the signal presented as largely unsaturated positive contrast (*i.e.* white). Because a SPIO nanoparticle concentration of 18.67 pg per cell provided a strong and localized signal by μCT, we chose this particular labeling concentration for our animal implantation experiments.

SPIO nanoparticle-labeled progenitor cells were incorporated in fibrin sealant and injected into the AV groove of Lewis rat hearts for 1 year. Because we were primarily interested in long-term tracking of transplanted cells, we imaged these animals at 6 months and 1 year following surgery. We were able to locate the cells both *in vivo* and *ex vivo* using MRI (Figure 2) and μCT (Figures 3 and 4). For the μCT experiments, we presented results from the rat heart that contained the fewest number of labeled cells localized to a discreet region within the implant to demonstrate the clarity and precision of this technique in identifying small numbers of cells. Imaging results were confirmed by extensive histological examination of serial tissue sections from the same heart (Figure 5) as well as from the other implanted hearts (Figures 5 and 6). Importantly, these experiments mimicked a preclinical cell delivery study where it would be critical for a label to remain intact for a period of months or years. The rare appearance extracellular SPIO particles observed in histological sections was presumed to be due to particles that remained attached to the plasma membrane of cells during the initial labeling procedure or represented unincorporated nanoparticles that were not removed during washing of the culture plates. Overall, our imaging experiments and histological analyses unequivocally demonstrate the utility of complementary MRI and μCT for long-term, non-invasive tracking of transplanted cells.

The present study should dispel the notion that imaging of SPIO-labeled cells over time using MRI (and μCT) is prone to excessive false positive or negative (*i.e.* type I and type II) errors because acquired signals may not accurately reflect the position and viability of cells *in situ* or the contrast provided by the label deteriorates after a few days or weeks [19]. To circumvent the release of iron particles from dying cells, ingestion of labeled cells by macrophages, and dilution of SPIOs by mitosis, we implanted Feridex-labeled cells incorporated in engineered tissues in the hearts of syngeneic Lewis rats. Although, we did not specifically enumerate cell death *in vivo*, viable myoblasts were easily detected for one year by histological examination. The cells used for these experiments were largely post-mitotic, which resulted in little diminishment of signal from dilution of intracellular iron oxide. In addition, transplanted myoblasts elicited a negligible immune response as measured at 6 months and 1 year.

In our earlier studies using a similar experimental protocol, we showed a modest inflammatory cell infiltration in the region of the implant that disappeared after a few weeks. This reaction did not significantly impact the retention of cells transplanted into the heart [23]. Additionally, the delivery of labeled cells by means of surgical placement of engineered tissues tends to avoid the high shear forces experienced by cells that are directly injected into the ventricular myocardium through small gauge needles. The Duploject system used for cell delivery has a very wide-bore fitting that greatly minimizes exposure of cells to excessive shear stress. Moreover, the engineered tissues were implanted into hearts that had not been injured prior to implantation (*e.g.* by myocardial infarction) [23], [28]. In short, the method of delivery, the anatomical position of the implant, and the health of the target organ all contributed to the survival of the progenitor cells over a long period of time [23], [29].

Despite the potential for the application of MRI and μCT to cell-based regenerative medicine studies, we recognize there are certain obstacles associated with routine use of these non-invasive imaging modalities. These are largely associated with the cost of performing repeated, multiple MRI and μCT scans in addition to limited access to this type of equipment. As far as clinical trials are concerned, exposure of humans to high levels of ionizing radiation from X-ray computed tomography is undesirable, particularly in the pediatric patient population. On the other hand, the continuing development of these and other imaging systems may result in greater availability and safety. As cell-based therapies move from preclinical testing using animal models to human trials, the need for serial evaluations of patients may also decline. Either way, if MRI and μCT availability increases in the research setting, the ability to combine information derived from multiple imaging modalities will represent a considerable opportunity for researchers interested in regenerative medicine.

## Conclusions

Here, we present data demonstrating the utility of SPIO nanoparticles as a long-term, non-genetic label for non-invasive cell tracking. We identified the detection limits for this contrast agent using MRI and μCT and precisely localized labeled cells within engineered tissues implanted in the heart for up to one year. Detection of transplanted cells labeled with SPIO nanoparticles in animals and isolated hearts was found to be exceedingly sensitive and specific using a combination of these imaging modalities. We believe use of these complementary techniques has immediate relevance for preclinical testing of cell-based therapies [16].

## Supporting Information

Movie S1 **Whole animal, coronal MRI movie of SPIO-labeled cells implanted in a rat heart for 6 months.** The SPIO-labeled implant is the black spot located on the upper left-hand portion of the heart above the right ventricle and beneath the right atrium that does not disappear throughout the cardiac cycle. Other hypointense regions that are caused by blood flow typically do disappear as blood is pumped out of each chamber.
(AVI)

Movie S2 **Whole animal, short axis MRI movie of SPIO-labeled cells implanted in a rat heart for 6 months.** The SPIO-labeled implant is the black spot located on the upper left portion of the heart above the right ventricle and beneath the right atrium that does not disappear throughout the cardiac cycle. This movie corresponds to the still image shown in Figure 2B (left panel).
(AVI)

Movie S3 **Whole animal, volume rendered movie from serial μCT images of SPIO-labeled cells implanted in a rat heart for 1 year.** The implant is the small white object located within the thoracic cavity that can be easily identified within the rib cage. This movie corresponds to the histological sections depicted in Figure 5 (A to C) and closely approximates the size, shape, and location of heavily-labeled cells in the implant.
(MPG)

Movie S4 **Isolated heart, volume rendered movie from serial μCT images of SPIO-labeled cells implanted in a rat heart for 1 year.** The iron-labeled cells in the AV groove are depicted in red. As confirmed by histological examination, the positive signal emanates from a region between the right atrium and the aorta, above the right ventricle.
(MPG)
